# Leo program, a short multi-family skill-based psychoeducational program for caregivers of relatives living with a severe mental disorder: a retrospective pilot study

**DOI:** 10.3389/fpsyt.2024.1374540

**Published:** 2024-05-09

**Authors:** Louis-Ferdinand Lespine, Bénédicte de Martène, Blandine Zeltner, Bénédicte Chenu, Céline Dubien Berbey, Romain Rey

**Affiliations:** ^1^Center for Caregivers in Psychiatry of Lyon, Le Vinatier Hospital, Bron, France; ^2^Division for Clinical Research and Innovation, Le Vinatier Hospital, Bron, France; ^3^School of Medicine, UFR Simone Veil, Université Versailles Saint-Quentin-en-Yvelines (Paris Saclay University), Paris, France; ^4^Collectif Schizophrénies, Paris, France; ^5^Fondation FondaMental, Créteil, France; ^6^University Lyon 1, CNRS, INSERM, Lyon Neuroscience Research Center U1028 UMR5292, PSYR2, Bron, France

**Keywords:** caregivers, family psychoeducation, skills, severe mental disorder, burden

## Abstract

**Background:**

Caring for a relative with a severe mental disorder (SMD) is associated with high levels of burden and poor physical and mental health. There is a dire need for family psychoeducational programs that can be provided as early as possible. This manuscript describes the pilot testing of “Leo” a motivational-based psychoeducational program for caregivers of individuals with a SMD. The Leo program aims to provide caregivers with skills to best support their relative and to adopt self-care behaviors.

**Methods:**

We retrospectively analyzed medical records of caregivers who enrolled in a short, multi-family, skill-based psychoeducational program, consisting of eight 3-hour sessions over 8 weeks. Outcomes of interest included: i) adherence to the program, ii) satisfaction and perceived usefulness, and iii) pre-post changes in self-reported levels of depression (CES-D), burden (ZBI), and skills (10 Likert-scaled items). A network analysis was used to investigate the relationships between pre-post changes in self-evaluated skills and pre-post changes in burden and depression levels.

**Results:**

Over the 91 enrolled participants, 87 (95.6%) completed the program attending at least 5/8 sessions, 80.5% attending all sessions. Seventy-six caregivers fulfilled the questionnaires at baseline and after the program, and were included in the analysis. Although there was no evidence for significant change in self-reported depression levels (Cohen’s d=0.19, p=0.210), burden scores and all evaluated skills were improved post-intervention, with medium to strong effect size (Cohen’s ds from 0.47 to 0.87; p<0.001). Network output indicated that increased self-evaluated competence in 5 skills were associated with a global improvement in caregivers’ burden and/or depression scores. Post-intervention, 89.7% of caregivers were “very satisfied” and 82.1% found the program “extremely useful”.

**Conclusion:**

This pilot retrospective study shows high levels of satisfaction, perceived usefulness, and adherence to “Leo”, a short, multi-family, skill-based psychoeducational program with promising results in improving caregivers’ burden, self-evaluated competence in coping with caregiving demands and in self-care behaviors. This study provides preliminary insights into the mechanisms by which family psychoeducation might alleviate burden of care. A larger-scaled, controlled, randomized study with follow-up assessments is warranted to determine how burden, depression, and skills, as well as their inter-relationships, evolve over time.

## 1 Introduction

Support for individuals living with a severe mental disorder (SMD) is often provided by family caregivers. Since family caregivers fulfill multiple roles in the care of their relative, they are key actors for promoting their recovery (1). However, caring for a relative with a SMD often results in high levels of *burden*, which entails the physical, psychological, social and financial consequences borne by family caregivers (2, 3). As compared to the general population, family caregivers are more likely to experience social isolation, low quality of life, and poor physical and mental health (2–5). In turn, this increased distress and burden impede caregivers’ ability to provide help that their relative may need to achieve recovery goals.

In this light, various interventions have been created to support caregivers, empower them and prevent burden development. Among such resources, *family psychoeducation* (FPE) is an evidence-based practice (6) and an important part of optimal treatment for people with psychotic, bipolar or depressive disorders (7–9). FPE aims to provide caregivers with knowledge and skills to better support their relative with a SMD as well as to maintain their own well-being. FPE is one of the most effective psychosocial interventions for relapse and re-admission prevention in schizophrenia (10) with effect sizes equating to those obtained by antipsychotic medication (1). FPE is also associated with improved adherence to medication (11), better social functioning and increased employment (1, 11). Although less studied, positive outcomes on family have been reported including reduction in burden and psychological distress, and improvement in knowledge, coping and family functioning (12–15).

Although meta-analyses and international guidelines advocate that FPE should be provided early and systematically (9, 10, 16–20), it remains insufficiently available. For instance, among the 4.5 million French caregivers in psychiatry, only 3% have benefited from FPE, provided on average 10 years after the disease onset (21, 22). In France, FPE is mainly provided through long-lasting, diagnostic specific programs. Although highly efficient, such programs remain scarce and many caregivers in early settings are reluctant to attend them. Therefore, further development of brief FPE and research into its benefits is warranted (23, 24).

In a participatory approach with family associations (i.e., peer-led organizations supporting caregivers of individuals living with a SMD), we recently co-designed and implemented in our psychiatry department a short, multi-family, multi-diagnostic, FPE program called “Leo”. This program is designed to provide caregivers with day-to-day skills based on two complementary motivational approaches: Motivational Interviewing (MI) and the Zurich Resource Model (ZRM). MI is a well-established strategy for facilitating behavior (25). It includes a set of communication tools designed to decrease defensiveness and rigidity (26). Although originally developed for health professionals, non-professionals have been trained to MI with positive effects on risky health behaviors (27, 28). Training caregivers to MI is considered a promising approach to improve their relationships with a relative living with recent onset schizophrenia (29) or early psychosis (24). The ZRM, a self-management training concept, was incorporated to help caregivers durably adopt self-care behaviors. The ZRM relies on the development of motto-goals to activate a person’s resources towards complex situations while activating intrinsic motivation (30, 31). The ZRM has shown positive effects on stress and affect regulation abilities in various target groups such as subjects with eating disorders or asthma and individuals at risk for burnout syndrome (32–35).

The aim of this retrospective pilot investigation, based on a single-group pre-post quasi-experimental design, was to assess the satisfaction, adherence rate and potential effectiveness of the Leo program on self-reported levels of burden, depression, and skills, among caregivers of a relative living with a SMD.

## 2 Materials and methods

### 2.1 Participants

From March 2021 to April 2023, a total of 91 caregivers participated in the Leo program which was performed 10 times. The participants of this study met the following criteria: i) relatives of individuals living with a severe mental disorder (first episode of psychosis, schizophrenia or schizoaffective disorder, bipolar disorder, major depressive disorder, anxiety disorder, borderline personality disorder, according to the Diagnostic and Statistical Manual (DSM-5), based on the consensus rating by the psychiatrists in charge). Noteworthily, some caregivers didn’t know the precise diagnosis of their relative (when the onset of the disorder was recent or when the diagnosis had changed over time or because their relative had not shared it with them). In such a situation, caregivers could still participate in the program when they reported that their relative was receiving/had received substantial psychiatric care, ii) relatives of individuals who were inpatients or outpatients, iii) aged 18 years or older. There were no exclusion criteria.

### 2.2 The family psychoeducation program

The Leo program was co-designed with members of family associations in a participatory research approach. It consists of eight 3-hours-sessions, over the course of 8 to 10 weeks, in groups of 8 to 10 participants (caregivers). The first session is led by a psychiatrist and consists in a theoretical course on medical knowledge about SMD. Sessions 2 to 5 provide competences to better support one’s relative. During these sessions, caregivers practice motivational interview techniques: asking open-ended questions, making affirmations, using reflections, summarizing, giving advice in order to facilitate behavioral changes. Caregivers are also trained to express dissatisfaction, to set limits or optimize a request. During the last 3 sessions (sessions 6 to 8), caregivers are trained to the self-management ZRM technique: they progressively build their own motto-goal to sustainably adopt a new attitude toward difficult situations or to approach a personal goal. The program is led by two professionals (typically a psychologist and a psychiatric nurse) present during the entire program. Three months after the eighth session, caregivers are invited to attend an optional session which allows them to re-train the competences previously taught in the form of solving concrete situations. To promote competence acquisition, a significant proportion of time is devoted to active training. Indeed, sessions 2 to 8 are organized as follows: 1 hour of theoretical input and 2 hours of practical training. During the program, caregivers are regularly divided in groups of 4, allowing simulation-based training techniques such as peer role-play and simulated patients and fostering peer support and empowerment.

### 2.3 Measures and procedure

Caregivers who registered in the psychoeducational program completed self-reported questionnaires evaluating their levels of depression, burden, and perceived competences, before the first (pre-questionnaire) and immediately after the last (post-questionnaire) session. Socio-demographics (age, gender), relationships to the relative, and relative’s diagnosis were collected before the first session. Measures of satisfaction and perceived usefulness (introduced after the program started, in late 2022) were evaluated immediately after the last session.

#### 2.3.1 Depression

Depression symptoms were assessed using the 20-item Center of Epidemiological Studies - Depression (CES-D) scale (36, 37), a self-report measure where participants indicate how often over the past week various statements such as ‘I felt lonely’ or ‘I felt sad’ applied to them. Each item is rated on a 4-point Likert scale that ranges from 0 (‘rarely’ or ‘none of the time’) to 3 (‘most of the time’), total scores ranging from 0 to 60. Cronbach’s α of the CES-D in the present study was 0.92.

#### 2.3.2 Burden

Caregiving burden was assessed using the 22-item version of the Zarit Burden Interview (ZBI) (38, 39), a self-report measure where individuals indicate how often various statements such as ‘you feel that your relative asks for more help than he/she needs’ or ‘you feel embarrassed about your relative’s behavior’ applied to them. Each item is rated on a 5-point Likert scale that ranges from 0 (‘never’) to 4 (‘nearly always’), total scores ranging from 0 to 88. Cronbach’s α of the ZBI in the present study was 0.90.

#### 2.3.3 Competences/skills

The 10 main competences provided during the program (listed in **Table 1**) were divided into two subgroups: namely, *to better support one’s relative* (competences 1 to 6) and *to adopt self-care behaviors* (competences 7 to 10). Self-estimation of the 10 competences was assessed, each competence being rated from 0 (‘none’) to 10 (‘complete’).

**Table 1:** Competences self-evaluated at pre- and post-intervention.

On the scale of 0 (‘none’) to 10 (‘complete’), how would you rate your competence…	
C1	To communicate with your relative
C2	To set limits
C3	To make a request to the care team
C4	To make a request to your relative
C5	To spot the warning signs of a crisis
C6	To communicate with your relative in the presence of delusions or hallucinations
C7	To identify your signs of exhaustion
C8	To locate your resources
C9	To identify your needs
C10	To sustainably adopt new behaviors

#### 2.3.4 Satisfaction and perceived usefulness

Participants were asked to rate their level of satisfaction on a 4-point Likert scale as ‘dissatisfied’, ‘neither satisfied nor satisfied’, ‘satisfied’, or ‘very satisfied’. Similarly, perceived usefulness of the program was rated on a 5-point Likert scale as ‘not useful’, ‘slightly useful’, ‘moderately useful’, ‘very useful’, or ‘extremely useful’.

### 2.4 Ethical consideration

The protocol was approved by Le Vinatier hospital review board prior to data extraction (CEREVI/2024/015). All caregivers gave consent for the use of their health data in the present study, which was approved by the French Data Protection Commission (No. 17372720).

### 2.5 Statistical analyses

Data collected from the caregivers who attended the 10 Leo programs organized between March 2021 to April 2023 were collapsed for the present analysis. Item-level missing data for the CES-D, ZBI, and competences/skills, when occurred, ranged from 1.3% (n=1) to 10.4% (n=8), and were replaced by the median derived from same-scale values of the corresponding participant (**Supplementary Tables 1**, **2**). Pre-post changes in total scores of the CES-D and the ZBI, and each competences/skills were evaluated using two-tailed paired *t*-tests with Bonferroni correction, effect size being calculated using Cohen’s *d*. Because deviation from normality, when occurred (for competences/skills), was minimal, *t*-tests were preferred over less-powered non-parametric alternative. The McNemar test with the mid-p-value approach (40) was used to determine the significance of pre-post differences in proportions of caregivers who had a CES-D score ≥ 16 (41), and a ZBI score ≥ 41 (moderate-to-severe burden). Exploratory network analysis was used in order to get insight into the relationships between i) pre-post changes in competences and ii) pre-post changes in ZBI (network A) or CES-D (network B) scores. Network analysis includes graphical representations of the relationships between variables represented by ‘nodes’ connected by ‘edges’ (42). Networks were estimated using the *estimateNetwork* function in the R package *bootnet* (43) with the “EBICglasso” method computing a Gaussian graphical model with the graphical LASSO (44) and extended Bayesian information criterion EBIC; (45) for model selection. In Gaussian graphical models, the parameters (i.e., edges) represent the association among two variables after conditioning on all other variables in the network (i.e., partial correlations). The LASSO (‘least absolute shrinkage and selection operator’ (46)) is a regularization technique allowing parameters to be zero, resulting in a sparse network. The strength of the penalty of the LASSO is controlled by a parameter λ, selected using the EBIC which itself has a tuning parameter γ (we used the default value 0.5).

## 3 Results

### 3.1 Adherence, characteristics of the sample and satisfaction/usefulness

**Figure 1:**
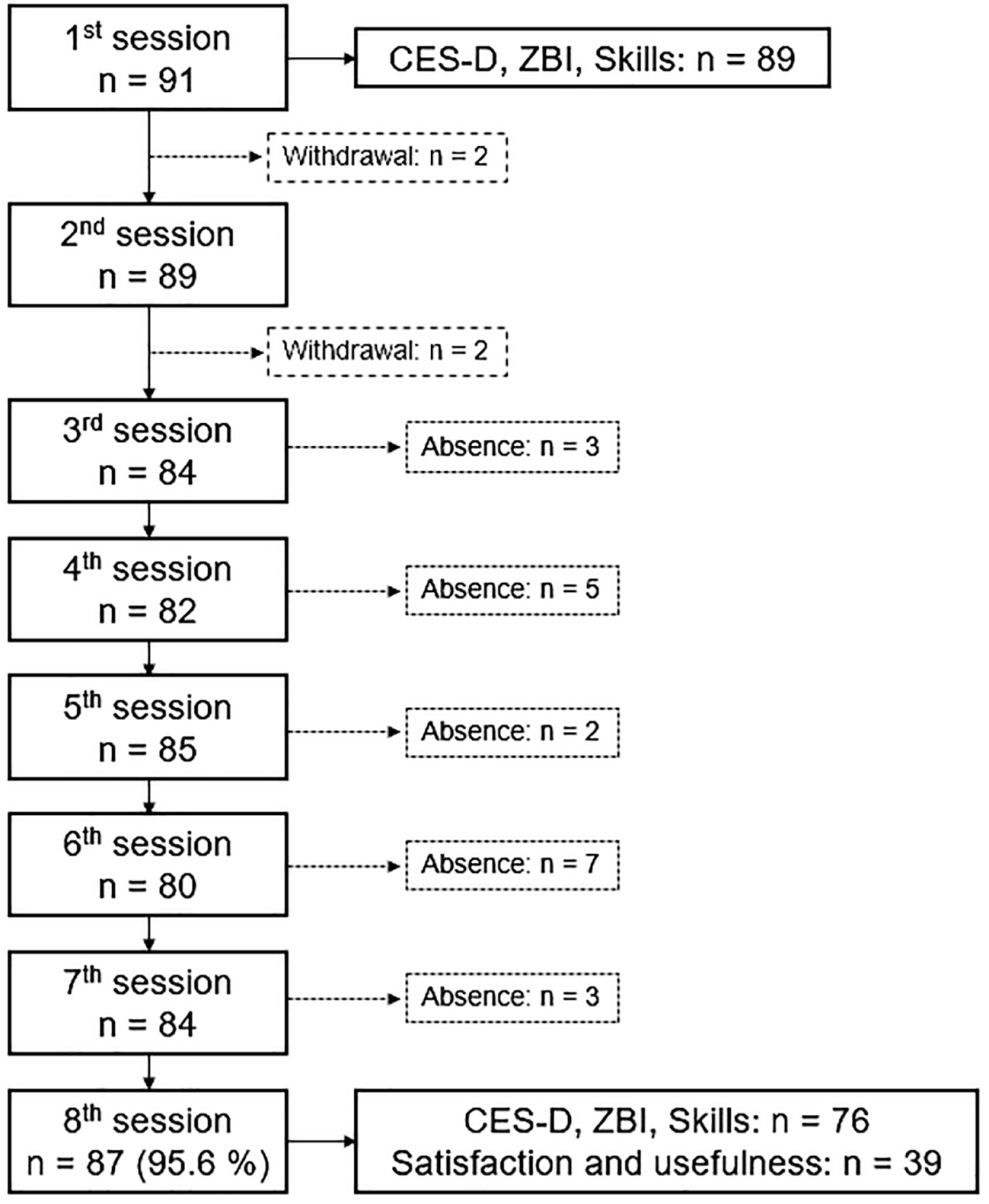
Flowchart of participants.

Flowchart of participants is shown in **Figure 1**. Over the 91 participants, 87 (95.6%) completed the program, attending at least 5/8 sessions, 98.9% attended 6/8 sessions, 95.4% attended 7/8 sessions, and 80.5% attended all sessions. Although some caregivers spontaneously reported impediment or unavailability, reasons for absence were not systematically collected. Notably, when a participant missed a session, he was systematically offered the opportunity to catch up on the content through a supplementary short individual session. When asked about reasons for quitting the program, participants (n=4) reported either i) feeling mentally exhausted (so much that they felt unable to continue the program) or ii) unready for a group experience (it was emotionally difficult to be confronted with others). Among the remaining 87 caregivers, 76 fulfilled the pre- and post-intervention questionnaires and were included in the analysis. More precisely, 2 (2.2%) participants did not return the pre-intervention questionnaires, and 9 further participants (9.9%) did not return the post-intervention questionnaires (or were not exploitable).

The characteristics of the 76 subjects included in the analysis are presented in **Table 2**. The mean age of caregivers was 58.5 years (SD=10.6 years). Most caregivers were women (76.3%) and were caring for their child (72.4%). Relatives’ psychiatric diagnosis included schizophrenic or schizoaffective disorder (39.5%), bipolar disorder (23.7%), a first episode of psychosis (10.5%), borderline personality disorder (9.2%), major depression disorder (2.6%), and anxiety disorder (2.6%). The diagnosis was unknown or unspecified for 11.8% of caregivers.

**Table 2:** Sample characteristics (n = 76).

Age, mean (SD)	
	58.5 (10.6)
Gender, n (%)	
Women	58 (76.3)
Men	18 (23.7)
Relationship to the relative, n (%)	
Parent/Step-parent	55 (72.4)
Partner	9 (11.8)
Sibling	8 (10.5)
Child	2 (2.6)
Other	2 (2.6)
Relatives’ diagnosis, n (%)	
Schizophrenic or schizoaffective disorder	30 (39.5)
Bipolar disorder	18 (23.7)
First episode of psychosis	8 (10.5)
Borderline personality disorder	7 (9.2)
Major depressive disorder	2 (2.6)
Anxiety disorder	2 (2.6)
No diagnosis/No knowledge of diagnosis	9 (11.8)

**Figure 2:**
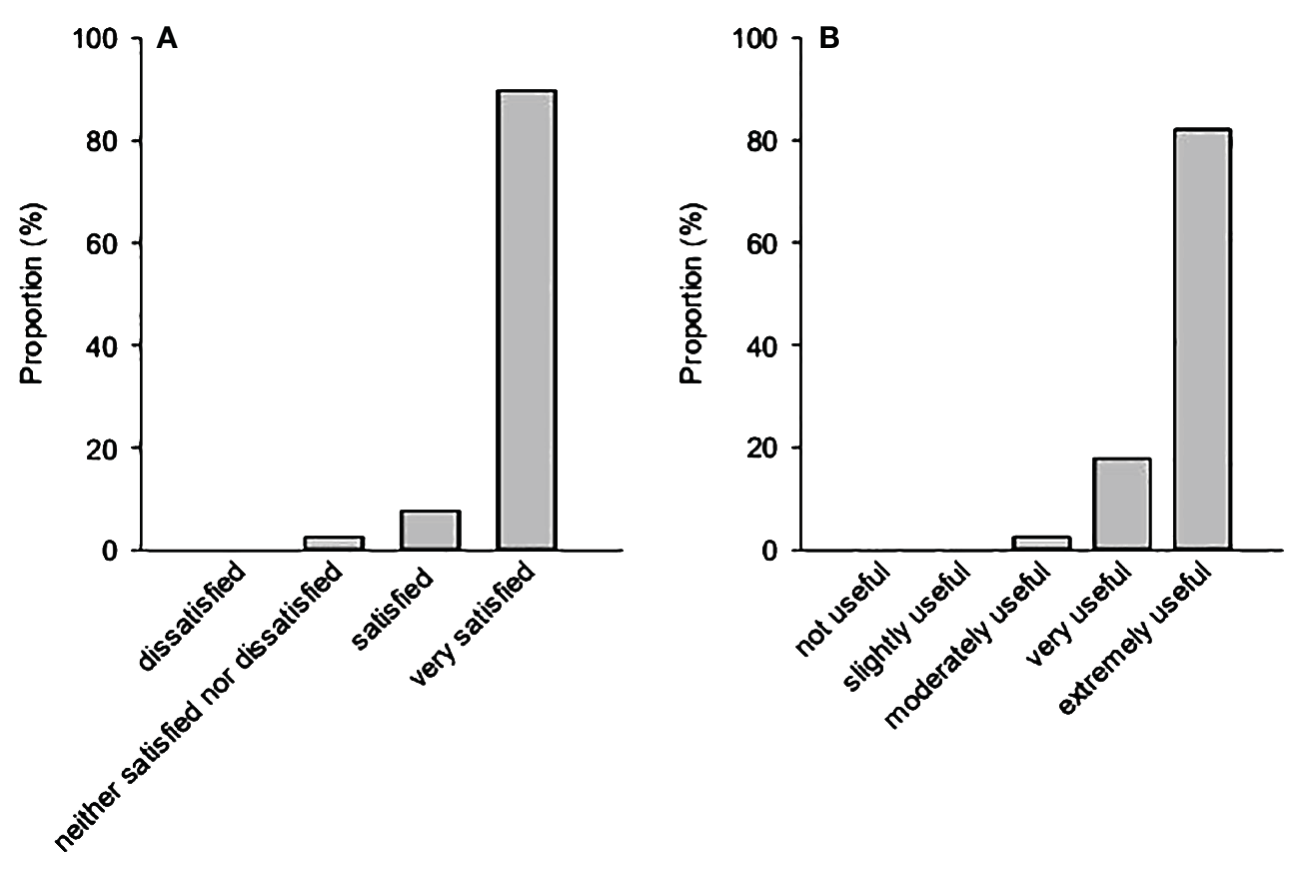
**(A)** Level of satisfaction and **(B)** perceived usefulness of the program, assessed immediately after the program (last session).

Finally, most caregivers — for whom the satisfaction and usefulness questionnaires were available (n= 39; 51.3%) — were “very satisfied” (89.7%) and found the program “extremely useful” (82.1%). None of the participants were either “dissatisfied” or rated the program as “not useful” (**Figure 2**).

### 3.2 Pre-post changes in depression, burden, and competences

Results are reported in **Table 3** and **Supplementary Table 3**. Although CES-D scores slightly decreased from baseline to post-intervention, there was no evidence for a statistical significance (*p*=0.103, *d*=0.19). Forty caregivers (52.6%) had a CES-D score ≥16 at baseline, 35 (46.1%) after the intervention (McNemar test, *p*=0.210). Likewise, there was no evidence (McNemar test at *p*=0.057) for differential proportions of subjects with a ZBI score ≥41 (moderate-to-severe burden) between baseline (n=45; 59.2%) and post-intervention (n=38; 50.0%). However, ZBI scores showed significant improvement from baseline to post-intervention, with moderate effect size (*p*<0.001, *d*=0.52). Competences/skills moderately or strongly improved post-intervention as compared to baseline, Cohen’s *d*s ranging from 0.47 to 0.87 (all *p*-values <0.001).

**Table 3:** Pre-post change in participants’ self-evaluated depression, burden, and competences (n=76).

	Baseline M (SD)	Post-intervention M (SD)	*t*-test_(75)_ (*p*-value)	Cohen’s *d*
Depression				
CES-D	18.1 (10.2)	16.8 (10.0)	1.651 (0.103)	0.19
Burden				
ZBI	41.6 (14.2)	37.2 (15.2)	4.564 (<0.001)	0.52
Competences (ranged by effect size)				
C10	5.0 (2.1)	6.9 (1.3)	-7.569 (<0.001)	0.87
C6	4.0 (2.4)	6.1 (2.0)	-7.331 (<0.001)	0.84
C9	5.5 (1.9)	7.1 (1.2)	-6.690 (<0.001)	0.77
C5	5.7 (2.3)	7.1 (1.5)	-6.479 (<0.001)	0.74
C4	5.5 (2.1)	6.8 (1.6)	-6.121 (<0.001)	0.70
C1	5.7 (1.8)	6.8 (1.3)	-6.090 (<0.001)	0.70
C8	5.5 (2.1)	7.1 (1.4)	-5.496 (<0.001)	0.63
C2	5.1 (2.2)	6.4 (1.6)	-5.142 (<0.001)	0.59
C7	5.8 (2.0)	7.2 (1.5)	-4.576 (<0.001)	0.53
C3	6.0 (2.7)	7.2 (1.9)	-4.094 (<0.001)	0.47

Except for CES-D, t-tests resulted in a p-value lower than the Bonferroni-corrected alpha (0.05/12 = 0.0042).

C1: To communicate with one’s relative. C2: To set limits. C3: To make a request to the care team. C4: To make a request to one’s relative. C5: To spot the warning signs of a crisis. C6: To communicate with one’s relative in the presence of delusions or hallucinations. C7: To identify one’s signs of exhaustion. C8: To locate one’s resources. C9: To identify one’s needs. C10: To sustainably adopt new behaviors.

### 3.3 Network output

**Figure 3:**
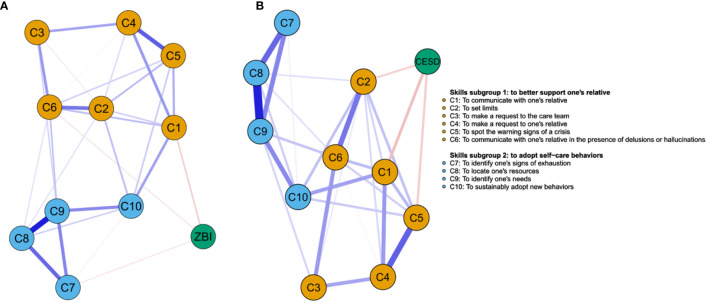
**(A)** Network displaying the relationships between pre-post change in competences and pre-post change in burden. **(B)** Network displaying the relationships between pre-post change in competences and pre-post change in depression. Nodes represent pre-post change in self-evaluated depression, burden, and competences. Blue and red edges represent positive and negative partial correlations between nodes, respectively. The thickness of the line indicates the strength of the association (i.e., the edge weight).

Estimated networks are presented in **Figure 3**. The corresponding partial correlations matrices are reported in **Supplementary Table 4**. Pre-post changes in competences/skills were inter-related. For instance, better skills at “identifying one’s own signs of exhaustion” (C7), “one’s resources” (C8) and “one’s needs” (C9) were closely related. Improvement in “spotting the warning signs of a crisis” (C5) was primarily associated with better skill at “making a request to one’s relative” (C4), the latter being also associated with improvement in “communicating with them” (C1). Better skill at “communicating with one’s relative in the presence of hallucinations or delusions” (C6) was linked to improvement in “setting limits” (C2) and “making a request to the care team” (C3). Better competence at “adopting new behaviors” (C10) was primarily associated with improvement at “identifying one’s needs” (C9) and “communicating with one’s relative” (C1). Global improvement in self-reported burden and depression (i.e., decrease in total score) were both primarily associated with better skill at “communicating with one’s relative” (C1). Improvement in burden scores was further associated with better competence at “communicating with one’s relative in the presence of delusions or hallucinations” (C6), and at “identifying one’s signs of exhaustion” (C7). Improvement in depression scores was further associated with better competence at “setting limits” (C2), and “spotting the warning signs of a crisis” (C5).

## 4 Discussion

In the present pilot study, we retrospectively analyzed data from caregivers of individuals living with a SMD who enrolled in a short, multi-family, skill-based FPE program. Participants showed a high level of satisfaction and adherence to the program. Caregivers’ self-evaluated skills were all significantly improved post-intervention. Following the FPE program, we report preliminary evidence of a significant overall improvement in caregivers’ burden whereas we found no evidence for a significant change on self-reported depression.

Participants’ satisfaction with the Leo program was high. Indeed, 89.7% of the participants who completed the post-intervention satisfaction survey were “very satisfied”, and 82.1% found the program “extremely useful”. Of note, none of the participants were either “dissatisfied” or rated the program as “not useful”. Furthermore, we report a high adherence rate to the program, since 95.6% of the participants achieved the Leo program, attending at least 5 sessions, most of them (80.5%) attending all sessions. Altogether, findings from this pilot study support the feasibility of delivering a short, multi-family, skill-based FPE program to caregivers of subjects living with various SMD. This is possibly due to our prioritization of feasibility in the design of the intervention. Indeed, the program is accessible to all caregivers caring for a relative living with a SMD, without any constraint of caring duration or diagnosis (even if the diagnosis is not yet known). Furthermore, the high level of commitment probably reflects our responsiveness to caregivers requesting a brief intervention that does not require the participation of the relative living with a SMD. Altogether, such characteristics allow all interested caregivers to benefit from the Leo program without any delay or constraint, thus preserving a high level of motivation.

In the present study, caregivers’ self-evaluated competences were all significantly improved post-intervention as compared to baseline with medium to strong effect sizes. Importantly, caregivers reported increased sense of competence in both subgroups of skills (i.e., *to better support one’s relative* and *to adopt self-care behaviors*). This is consistent with the previous report that a short, skill-building FPE program increased self-efficacy and self-care behaviors of caregivers of individuals with dementia (47). Our findings are thus preliminary evidence supporting the efficiency of a short, skill-based FPE program to improve competence of caregivers in psychiatry. This is consistent with a previous RCT reporting the beneficial effect of another short, skill-based FPE program on caregiver’s self-efficacy (24). In contrast, a previous RCT by Kulhara et al. evaluating a long FPE program reported no significant improvement in caregiver-coping immediately after the program (48). Different factors may explain the discrepant results. First, the two studies explored different outcomes since caregivers’ competence and caregivers’ coping strategies are different constructs (49). Second, the two FPE programs differed regarding key characteristics: especially the number of sessions devoted to skills training (2 sessions in Kulhara et al.’s program vs. 7 sessions in the Leo program) or the education techniques used during the FPE. Indeed, skill training is a more difficult educational objective as compared to knowledge improvement. Therefore, simulation-based training techniques such as peer role-play and simulated patients were incorporated in the Leo program to improve skills learning. In simulation-based education, the educational objective of role-play training is to rehearse situations to improve learner’s abilities to face with similar situations in daily life (50). Since simulation-based training is considered an efficient teaching technique to promote skills learning (51), it is possible that devoting significant time to simulation during the Leo program helped to improve caregivers’ sense of competence.

As expected, the caregivers included in the present study reported high levels of depressive symptoms and burden at baseline. Indeed, 52.6% of them were possibly depressed (CES-D score ≥ 16) while 59.2% exhibited a moderate-to-severe burden (ZBI score ≥ 41). Notably, the percentage of caregivers with a CES-D score ≥ 16 was higher than the ones previously reported in caregivers of subjects with bipolar disorder or schizophrenia (respectively, 22-to-33% and 42%) (52–55). In contrast, the rate of caregivers with a ZBI ≥ 41 was consistent with those (ranging from 30 to 68%) previously observed in caregivers of patients with SMD (56–59). Altogether these results confirm that caregivers in psychiatry are a vulnerable population characterized by a high risk of depression as compared to the general population (2, 3) and therefore require dedicated interventions such as FPE.

In the present study, we found no evidence for a significant difference in self-reported depression score immediately after the Leo program. Remarkably, a recent meta-analysis reported no significant effect of FPE combined with skills training on patient’s relapse at 6 months whereas, a significant reduction was observed at 12 months and further (17). This suggests that a delay might be necessary to observe the beneficial effects of this type of skill-based FPE. Indeed, skills are often more complex to implement in daily-life as compared to knowledge. Caregivers may implement them gradually after the FPE which could explain why beneficial effects are observed only after a delay. In psychiatry, although previous studies of short, knowledge based FPE programs have reported a significant reduction of caregivers’ depression immediately after the intervention (60) or at 6 months (61), unfortunately, we are not aware of any previous studies investigating the effect of a short, skill-based FPE program on caregivers’ depression. Further studies investigating the impact of skill-based FPE on caregivers’ depression are needed and should explore caregivers’ outcomes at various time points after FPE.

In contrast, a significant decrease of caregivers’ burden was observed after the intervention. Indeed, ZBI scores showed significant improvement from baseline to post-intervention, with a medium effect size. This is in accordance with previous pilot studies reporting a significant decrease in family burden immediately following a FPE program (60, 62). Similarly, two randomized controlled trials of FPE programs reported that skill-based FPE was associated with lower levels of family burden albeit at 6 and 12 months following the intervention (63, 64). Notably, the differential proportions of subjects with a ZBI score ≥ 41 (moderate-to-severe burden) between baseline (59.2%) and post-intervention (50.0%) was marginally significant (p=0.057). Family burden is a much broader and subjective concept as compared to depression. It involves not only physical and psychological impact but also sense of losing control (65). For caregivers, increased caregiving-related skills enhance self-competence in challenging difficulties (66). In this light, it is possible that the decrease of caregivers’ burden observed following the Leo program was related to the significantly improved sense of competence reported by caregivers after the intervention.

Improved caregiving skills are associated with better health outcomes for caregivers of subjects living with a SMD (67). Such findings have informed the development of psychoeducational interventions to support caregivers. However, the existing models of FPE greatly differ from one another. Although some criteria regarding the “key ingredients” underlying FPE efficiency have emerged (e.g., the type and number of participants, format, frequency of sessions, duration, location, teaching techniques), they are not precise enough when it comes to specify the content of the FPE sessions (coping, communication, problem solving) (23). There is thus a dire need to identify more precisely which type of content underlies the effectiveness of FPE and should therefore be prioritized in FPE programs (68).

In this regard, increased self-evaluated competence in 3 skills were associated with an improvement in caregivers’ burden: namely, *to communicate with one’s relative*, *to communicate with one’s relative in the presence of delusions or hallucinations* and *to identify one’s signs of exhaustion*. Similarly, increased self-evaluated competence in 3 skills were associated with an improvement in self-reported depression: *to communicate with one’s relative*, *to set limits* and *to spot warning signs of a crisis*. Therefore, feeling more skilled in communicating with one’s relative was associated with an improvement in both self-reported burden and depression. Noteworthily, among the 10 skills provided during the Leo program, *to communicate with one’s relative* showed the strongest association with the improvement of both caregivers’ outcomes. More research is needed to confirm if these skills should be considered as “key ingredients” and, as such, should be systematically incorporated in FPE skill-based programs and prioritized among other contents (e.g., more time should be devoted to them). Identifying such “key ingredients” is of outmost importance to achieve well standardized psychoeducational programs with clear definitions of the content of interventions (69).

Noteworthily, competences from to the two subgroups (subgroup 1, *to better support one’s relative* and subgroup 2, *to adopt self-care behaviors*) were linked to an improvement in caregivers’ burden, while only competences from subgroup 1 were associated with an improvement in self-reported depression. Furthermore, numerous interrelationships between competences belonging to each of the two subgroups were observed in each network, supporting a potential synergistic effect. Especially, *to sustainably adopt new behaviors* (C10, subgroup 2) exhibited the greatest number of interrelationships with competences from another subgroup in both networks. Altogether, these preliminary findings suggest that both types of competences should be considered as “key ingredients” for FPE programs. Moreover, providing both type of competences within a single FPE program could be associated with an increased effectiveness on caregivers’ burden. Eventually, it should be underlined that out of the 10 competences incorporated in the Leo program after a careful selection process involving members of family associations, 5 were linked to an improvement in caregivers’ outcomes. This strongly supports the strategic relevance of co-designing the content of FPE programs in a participatory research approach involving the recipients of such programs (70, 71).

In regard to its pilot nature the present study has several limitations. Firstly, the sample size was small and there was no control group. Moreover, the single-center design of the present study may affect the representativeness of the included sample. Secondly, the post-intervention evaluation was realized immediately after the FPE program while existing literature has shown that improvement in the main outcomes is observed at 6 to 12 months (17). Thus, the long-term consequences of this short FPE program remain unclear. Thirdly, the satisfaction questionnaires were introduced after the program started and were available for only a subset of the participants. Our results can thus be subjected to sampling bias. Eventually, the quantitative measures of skills used in the pre- and post-intervention questionnaires were specifically developed for the evaluation of the FPE program; they were thus not validated. Indeed, validated questionnaires assessing skills of caregivers are lacking. In this regard, when evaluating FPE programs incorporating skills training, other authors have also relied on non-evaluated instruments (47) or questionnaires evaluating coping strategies (48). None of these latter questionnaires were precise enough to specifically explore the skills targeted by the Leo program, therefore we developed self-administered questionnaires with consideration of previous research on skills of caregivers in psychiatry (72). Furthermore, to improve content validity, the questions were designed to explore the psychoeducational objectives of the Leo program. Such questionnaires are routinely used in FPE program research (47), although their accuracy has been questioned as compared to objective measures (73). Notably, a good agreement between self-administered evaluation and objective measures of performance was reported, supporting self-administered questionnaires as valid tools to assess performance on specific learning objectives (74).

In light of these limits, a larger, multi-center, randomized controlled study should be undertaken to confirm the present preliminary results and to explore caregivers’ and relatives’ outcomes in the medium and long-term. Eventually, health economic outcomes should be explored, since the efficiency of psychoeducation is central in making it an attractive intervention for managers and policy makers. As a well-standardized FPE program with clear definitions of the content of the interventions, Leo is a promising intervention for researchers planning to develop evidence-based programs for caregivers.

## 5 Conclusion

In addition to showing high levels of participant satisfaction and adherence to the FPE program Leo, this pilot study provides preliminary evidence that caregivers significantly improved burden as well as self-evaluated competence in supporting their relative and in self-care behaviors immediately after the intervention. In light of its brief format and large accessibility (no constraint as to the caring duration or diagnosis of the relative), the Leo program is expected to be easily disseminated within the mental health system. For the field of FPE, the present results have several implications. They support the feasibility and efficiency in routine care of a short, multi-family, multi-diagnostic, skill based FPE program for caregivers of subjects living with a SMD. The present research also provides insights into the critical contents through which FPE might alleviate burden of care. Eventually, these results strongly support the relevance of engaging caregivers in the coproduction of FPE programs. More well-standardized FPE programs with clear definitions of the content of the interventions should be developed. Additional randomized controlled studies are needed to confirm the present results and to explore caregivers’, relatives’ and health economic outcomes in the medium and long-term.

## Data availability statement

The raw data supporting the conclusions of this article will be made available by the authors, without undue reservation.

## Ethics statement

The studies involving humans were approved by the Comité d’Éthique Recherche du Vinatier (CEREVI)/Centre Hospitalier Le Vinatier. The studies were conducted in accordance with the local legislation and institutional requirements. The ethics committee/institutional review board waived the requirement of written informed consent for participation from the participants or the participants’ legal guardians/next of kin because as a retrospective study processing data drawn from personal medical files, the present research was granted MR-004 validation, which corresponds to the Commission Nationale Informatique et Libertés (CNIL) validation. In accordance with the French law for MR-004 standard, all subjects were informed regarding the research project and had the possibility of not consenting to the reuse of their health data for research.

## Author contributions

L-FL: Data curation, Methodology, Project administration, Validation, Writing – original draft, Writing – review & editing. BM: Investigation, Project administration, Supervision, Writing – original draft, Writing – review & editing. BZ: Investigation, Writing – original draft, Writing – review & editing. BC: Investigation, Writing – original draft, Writing – review & editing. CB: Data curation, Writing – original draft, Writing – review & editing. RR: Conceptualization, Investigation, Project administration, Resources, Supervision, Validation, Writing – original draft, Writing – review & editing.
