# Supplementary material for: Leo program, a short multi-family skill-based psychoeducational program for caregivers of relatives living with a severe mental disorder: a retrospective pilot study
Source: Front Psychiatry. 2024 May 9;15:1374540. doi: 10.3389/fpsyt.2024.1374540 (PMC11112345; doi:10.3389/fpsyt.2024.1374540)
Supplement: Supplementary file 1 [file Table_1.docx]

Supplementary Material

Leo Program, a Short Multi-Family Skill-Based Psychoeducational Program for Caregivers of Relatives living with a Severe Mental Disorder: a Retrospective Pilot Study

**Supplementary Table 1.** Item-level missing values, n (%), in 76 caregivers.

| **CES-D** |  |  |  |  | **ZBI** |  |  |  |  | **Competences/Skills** |  |  |
| --- | --- | --- | --- | --- | --- | --- | --- | --- | --- | --- | --- | --- |
|  | Pre | Post |  |  |  | Pre | Post |  |  |  | Pre | Post |
| Item 1 | 2 (2.6) | 1 (1.3) |  |  | Item 1 | - | - |  |  | Item 1 | - | - |
| Item 2 | - | - |  |  | Item 2 | - | 1 (1.3) |  |  | Item 2 | - | - |
| Item 3 | - | - |  |  | Item 3 | - | - |  |  | Item 3 | 2 (2.6) | 2 (2.6) |
| Item 4 | 3 (3.9) | 2 (2.6) |  |  | Item 4 | - | - |  |  | Item 4 | 1 (1.3) | - |
| Item 5 | - | 1 (1.3) |  |  | Item 5 | 1 (1.3) | 1 (1.3) |  |  | Item 5 | - | 3 (3.9) |
| Item 6 | 2 (2.6) | - |  |  | Item 6 | - | - |  |  | Item 6 | 4 (5.2) | 8 (10.4) |
| Item 7 | - | - |  |  | Item 7 | - | - |  |  | Item 7 | 2 (2.6) | 1 (1.3) |
| Item 8 | 2 (2.6) | - |  |  | Item 8 | 1 (1.3) | 1 (1.3) |  |  | Item 8 | - | 2 (2.6) |
| Item 9 | 3 (3.9) | 1 (1.3) |  |  | Item 9 | - | - |  |  | Item 9 | - | 1 (1.3) |
| Item 10 | 1 (1.3) | 1 (1.3) |  |  | Item 10 | - | - |  |  | Item 10 | 1 (1.3) | - |
| Item 11 | 1 (1.3) | - |  |  | Item 11 | - | - |  |  |  |  |  |
| Item 12 | 2 (2.6) | 1 (1.3) |  |  | Item 12 | - | - |  |  |  |  |  |
| Item 13 | - | - |  |  | Item 13 | - | - |  |  |  |  |  |
| Item 14 | - | - |  |  | Item 14 | - | - |  |  |  |  |  |
| Item 15 | - | - |  |  | Item 15 | - | 1 (1.3) |  |  |  |  |  |
| Item 16 | 1 (1.3) | 1 (1.3) |  |  | Item 16 | - | - |  |  |  |  |  |
| Item 17 | - | - |  |  | Item 17 | - | - |  |  |  |  |  |
| Item 18 | - | - |  |  | Item 18 | - | 1 (1.3) |  |  |  |  |  |
| Item 19 | - | - |  |  | Item 19 | - | - |  |  |  |  |  |
| Item 20 | - | - |  |  | Item 20 | - | - |  |  |  |  |  |
|  |  |  |  |  | Item 21 | - | - |  |  |  |  |  |
|  |  |  |  |  | Item 22 | - | - |  |  |  |  |  |

**Supplementary Table 2.** Example of missing data imputation for competences/skills (participant n°38).

|  | Before imputation | After imputation |
| --- | --- | --- |
| C1 | 8 | 8 |
| C2 | 8 | 8 |
| C3 | 5 | 5 |
| C4 | 6 | 6 |
| C5 | 8 | 8 |
| C6 | Missing | **7** |
| C7 | 7 | 7 |
| C8 | 7 | 7 |
| C9 | 7 | 7 |
| C10 | 7 | 7 |
| **Median** | **7** |  |

**Supplementary Table 3.** Contingency 2 x 2 tables, n (%) in 76 caregivers.

| **CES-D ≥ 16^a^** |  |  | |  |
| --- | --- | --- | --- | --- |
|  |  | Post | |  |
|  |  | No | Yes | Total |
| Pre | No | 31 (40.8) | 5 (6.6) | 36 (47.4) |
|  | Yes | 10 (13.2) | 30 (39.5) | 40 (52.6) |
|  | Total | 41 (53.9) | 35 (46.1) | 76 (100.0) |
| **ZBI ≥ 41^b^** |  |  | |  |
|  |  | Post | |  |
|  |  | No | Yes | Total |
| Pre | No | 28 (36.8) | 3 (3.9) | 31 (40.8) |
|  | Yes | 10 (13.2) | 35 (46.1) | 45 (59.2) |
|  | Total | 38 (50.0) | 38 (50.0) | 76 (100.0) |

^a^ cut-off based on ref 42

^b^ moderate-to-severe burden

**Supplementary Table 4.** Matrix of regularized partial correlations. Edges that involved ZBI and CESD changes are highlighted in blue.

| **Network A: post-pre change in competences and burden (ZBI)** | | | | | | | | | | | |
| --- | --- | --- | --- | --- | --- | --- | --- | --- | --- | --- | --- |
|  | C1 | C2 | C3 | C4 | C5 | C6 | C7 | C8 | C9 | C10 | ZBI |
| C1 |  |  |  |  |  |  |  |  |  |  |  |
| C2 | 0.10 |  |  |  |  |  |  |  |  |  |  |
| C3 | 0 | 0.04 |  |  |  |  |  |  |  |  |  |
| C4 | 0.20 | 0.08 | 0.18 |  |  |  |  |  |  |  |  |
| C5 | 0.15 | 0.13 | 0 | 0.32 |  |  |  |  |  |  |  |
| C6 | 0.13 | 0.27 | 0.20 | 0.02 | 0.10 |  |  |  |  |  |  |
| C7 | 0 | 0 | 0 | 0 | 0 | 0 |  |  |  |  |  |
| C8 | 0 | 0.06 | 0 | 0 | 0 | 0.06 | 0.30 |  |  |  |  |
| C9 | 0 | 0 | 0.09 | 0 | 0 | 0.11 | 0.24 | 0.44 |  |  |  |
| C10 | 0.19 | 0.12 | 0 | 0 | 0.10 | 0 | 0.03 | 0.10 | 0.23 |  |  |
| ZBI | -0.10 | 0 | 0 | 0 | 0 | -0.06 | -0.05 | 0 | 0 | 0 |  |
| **Network B: post-pre change in competences and depression (CES-D)** | | | | | | | | | | | |
|  | C1 | C2 | C3 | C4 | C5 | C6 | C7 | C8 | C9 | C10 | CESD |
| C1 |  |  |  |  |  |  |  |  |  |  |  |
| C2 | 0.09 |  |  |  |  |  |  |  |  |  |  |
| C3 | 0 | 0.04 |  |  |  |  |  |  |  |  |  |
| C4 | 0.20 | 0.08 | 0.18 |  |  |  |  |  |  |  |  |
| C5 | 0.14 | 0.12 | 0 | 0.31 |  |  |  |  |  |  |  |
| C6 | 0.13 | 0.27 | 0.20 | 0.02 | 0.09 |  |  |  |  |  |  |
| C7 | 0 | 0 | 0 | 0 | 0 | 0 |  |  |  |  |  |
| C8 | 0 | 0.06 | 0 | 0 | 0 | 0.06 | 0.30 |  |  |  |  |
| C9 | 0 | 0 | 0.08 | 0 | 0 | 0.11 | 0.24 | 0.44 |  |  |  |
| C10 | 0.19 | 0.12 | 0 | 0 | 0.10 | 0 | 0.03 | 0.10 | 0.23 |  |  |
| CESD | -0.13 | -0.09 | 0 | 0 | -0.10 | 0 | 0 | 0 | 0 | 0 |  |

**Skills subgroup 1: *to better support one’s relative***

C1: to communicate with one’s relative

C2: to set limits

C3: to make a request to the care team

C4: to make a request to one’s relative

C5: to spot the warnings signs of a crisis

C6: to communicate with one’s relative in the presence of delusions or hallucinations

**Skills subgroup 2: *to adopt self-care behaviors***

C7: to identify one’s signs of exhaustion

C8: to locate one’s resources

C9: to identify one’s needs

C10: to sustainably adopt new behaviors
